# Increase in Bloodstream Infection Due to Vancomycin-Susceptible *Enterococcus faecium* in Cancer Patients: Risk Factors, Molecular Epidemiology and Outcomes

**DOI:** 10.1371/journal.pone.0074734

**Published:** 2013-09-19

**Authors:** Carlota Gudiol, Josefina Ayats, Mariana Camoez, M. Ángeles Domínguez, Carolina García-Vidal, Marta Bodro, Carmen Ardanuy, Mora Obed, Montserrat Arnan, Maite Antonio, Jordi Carratalà

**Affiliations:** 1 Department of Infectious Disease, Hospital Universitari de Bellvitge, Barcelona, Insitut d’Investigació Biomèdica de Bellvitge (IDIBELL), University of Barcelona, L’Hospitalet de Llobregat, Barcelona, Spain; 2 Department of Microbiology, Hospital Universitari de Bellvitge, Insitut d’Investigació Biomèdica de Bellvitge (IDIBELL), University of Barcelona, L’Hospitalet de Llobregat, Barcelona, Spain; 3 Department of Haematology, Hospital Duran i Reynals, Insitut d’Investigació Biomèdica de Bellvitge (IDIBELL), University of Barcelona, L’Hospitalet de Llobregat, Barcelona, Spain; 4 Department of Oncology, Hospital Duran i Reynals, Insitut d’Investigació Biomèdica de Bellvitge (IDIBELL), University of Barcelona, L’Hospitalet de Llobregat, Barcelona, Spain; 5 REIPI (Spanish Network for Research in Infectious Disease), Instituto de Salud Carlos III, Madrid, Spain; 6 CIBERes (CIBER de Enfermedades Respiratorias), Instituto de Salud Carlos III, Madrid, Spain; University Medical Center Utrecht, Netherlands

## Abstract

We conducted a prospective study to assess the risk factors, molecular epidemiology and outcome of bloodstream infection (BSI) due to *Enterococcus faecium* in hospitalized cancer patients. Between 2006 and 2012, a significant increase in vancomycin-susceptible *E. faecium* BSI was observed among cancer patients. Comparison of 54 episodes of BSI due to *E. faecium* with 38 episodes of BSI due to *E. faecalis* showed that previous use of carbapenems was the only independent risk factor for *E. faecium* acquisition (OR 10.24; 95% CI, 1.35-77.66). All *E. faecium* isolates were susceptible to glycopeptides, whereas 97% showed high-level resistance to ampicillin and ciprofloxacin. All 30 isolates available for genotyping belonged to the hospital-associated *E. faecium* lineages 17, 18 and 78. After 2009, most of the isolates belonged to ST117 (lineage 78). Patients with *E. faecium* BSI were more likely to receive inadequate initial empirical antibiotic therapy than patients with *E. faecalis* BSI, and time to adequate empirical antibiotic therapy was also longer in the former group. No significant differences were found between the two groups regarding early and overall case-fatality rates. Independent risk factors for overall case-fatality were current corticosteroids (OR 4.18; 95% CI, 1.34-13.01) and intensive care unit admission (OR 9.97; 95% CI, 1.96-50.63). The emergence of *E. faecium* among cancer patients is a concern since there are limited treatment options and it may presage the emergence of vancomycin-resistant enterococci. A rationale approach that combines infection control with antimicrobial stewardship.

## Introduction

Enterococci are part of the normal human microbial flora. Historically, the majority of invasive enterococcal infections were caused by *Enterococcus faecalis*, followed by *Enterococcus faecium* [1]. In recent decades, however, the epidemiology of invasive enterococcal infections appears to be changing worldwide, and a number of trends have been recognized, notably, the global emergence of enterococci as important nosocomial pathogens and the emergence of resistance to commonly used antimicrobial agents, including penicillins, aminoglycosides and glycopeptides [1].

An increase in the number of *E. faecium* strains in hospitals in different countries has been documented during the last decade [2-4]. These isolates had in common not only the antibiotic resistance traits (to ampicillin, quinolones and to glycopeptides in some cases) but also several virulence factors that might have contributed to the success of *E. faecium* as a leading nosocomial pathogen [4,5]. Although these strains were initially classified within a single clonal complex 17, it appears that the genetic diversity of this CC allows the classification of all isolates in three main lineages (17, 18, and 78), which is a more accurate representation of the recent evolution of these isolates[6]. 

Management of severe infections due to resistant enterococcal strains, especially *E. faecium*, has therefore become a therapeutical challenge. However, most of the reported experiences regarding enterococcal infections concern the general, non-immunocompromised population, and they mainly involve vancomycin-resistant strains [7-12]. Additionally, the majority of the studies published to date have been carried out in the United States, where the epidemiological situation is very different from that occurring in Europe [1,13]. Furthermore, information regarding bloodstream infection (BSI) due to *E. faecium* in immunosuppressed patients with cancer is particularly scarce [10,11,14,15]. Given the above, the aim of the present study was to describe the incidence and risk factors for vancomycin-susceptible *E. faecium* BSI in a large prospective cohort of cancer patients. We also aimed to ascertain the clinical features, antimicrobial susceptibility, genotypes and outcome of BSI due to *E. faecium* in this population.

## Materials and Methods

### Setting, patients and study design

We conducted a prospective observational study in a 200-bed cancer referral centre for adults in Barcelona, Spain. From 1 January 2006 to 30 September 2012 all hospitalized cancer patients and haematopoietic stem cell transplant recipients with at least one episode of BSI were included in the study. Information on baseline characteristics, clinical features, empirical antibiotic therapy and outcome was carefully recorded in a specific database.

All episodes of BSI due to vancomycin-susceptible *E. faecium* were compared with those caused by vancomycin-susceptible *Enterococcus faecalis* in order to identify the risk factors for ampicillin resistance acquisition and to assess differences in clinical features and outcome. We also compared patients who died with those who survived in order to identify risk factors for mortality.

All BSI episodes at our hospital are reported and followed up by an infectious disease physician. Changes in antimicrobial treatment and general management were advised when necessary.

### Ethics statement

This observational study was approved by the Institutional Review Board Comité Ético de Investigación Clínica del Hospital Universitari de Bellvitge (Ethics Committee of Clinical Research-Hospital Universitari de Bellvitge), with the following reference number PR 232/10. To protect personal privacy, identifying information of each patient in the electronic database was encrypted. Informed consent was waived by the Clinical Research Ethics Committee because no intervention was involved and no patient identifying information was included.

### Definitions

Neutropenia was defined as an absolute neutrophil count <500/mm^3^. Current corticosteroid therapy was recorded when a patient was receiving corticosteroids at the time of the BSI episode or in the previous month. Prior antibiotic therapy was defined as the receipt of any systemic antibiotic for >48 hours during the previous month. BSI was considered to be from an endogenous source in those patients with neutropenia in whom no other BSI sites were identified. In those patients without neutropenia, an unknown source was considered if an evident origin of the infection was not identified [16]. Shock was defined as a systolic pressure <90 mmHg that was unresponsive to fluid treatment or which required vasoactive drug therapy. Empirical antibiotic therapy was considered inadequate if the treatment regimen did not include at least one antibiotic active *in vitro* against the infecting microorganism. Early case-fatality rate was defined as death within 48 hours of the BSI episode. Overall case-fatality rate was defined as death by any cause within the first 30 days of the onset of BSI.

### Microbiological studies

Blood cultures were performed by standard methods. Two sets of two blood samples were drawn from patients with suspected bloodstream infection. Blood samples were processed by the BACTEC 9240 system (Becton Dickinson Microbiology Systems) with an incubation period of five days. Positive blood samples were sub-cultured onto chocolate agar. Identification and antibiotic susceptibility were performed using commercially available plates (MicroScan, Siemens), following the manufacturer’s instructions. The antimicrobial susceptibility of isolates was interpreted according to current Clinical Laboratory Standard Institute criteria [17].

Thirty *E. faecium* strains isolated between 2006 and 2012 from single bacteraemic patients were available for genotyping. Pulsed field gel electrophoresis (PFGE) was performed in all strains after *Sma*I restriction of chromosomal DNA, as previously described [18]. PFGE patterns were interpreted both by visual inspection, using the criteria of van Belkum et al. and by analysis with the FINGERPRINTING TM II software, version 3.0 (BioRad Laboratories, Inc., Madrid, Spain) [19]. Dendrograms were constructed using Dice coefficients, with optimization and band position tolerance being set to 0.5% and 1% respectively. A similarity coefficient of 80% was selected to define the patterns.

Multilocus sequence typing (MLST) was conducted on 17 representative strains of each *Sma*I-PFGE type, as described by Homan et al. [20]. Sequence types (STs) were assigned according to the *E. faecium* MLST database (http://efaecium.mlst.net).

### Statistical analysis

Continuous variables were compared by means of the Mann-Whitney U test and *t*-test. Qualitative variables were compared using the chi-square test, and odds ratios and 95% confidence intervals were calculated. Multivariate conditional logistic regression analysis of factors potentially associated with *E. faecium* acquisition and mortality included all statistically significant variables in the univariate analysis, sex and age, and all clinically important variables regardless of whether they were statistically significant or not [21]. This analysis was performed with the stepwise logistic regression model of the SPSS software package (SPSS v. 17).

## Results

During the study period 1287 consecutive episodes of BSI were recorded. Of the 550 (42.5%) episodes caused by Gram-positive bacteria, 105 were due to enterococci (19%). Thirteen episodes of enterococcal BSI were not included in the study because they were caused by species other than *E. faecium* or *E. faecalis* ( *E* *. gallinarum* 6, *E* *. casseliflavus* 2, *E. avium* 2, *E* *. hirae* 1, *E. durans* and *E. raffinosus* 1). Thus, 54 episodes of BSI caused by *E. faecium* and 38 by *E. faecalis* were finally included in the study. Four patients with two episodes of enterococcal BSI were included since they were considered to present different episodes, separated by at least four weeks.

The incidence of *E. faecium* BSI increased significantly over time (22 episodes/126610 admissions from 2006 to 2009 vs 32 episodes/80586 admissions from 2010 to September 2012; *p*=0.002). By contrast, the incidence of *E. faecalis* BSI remained stable over time (p=0.215).

Table 1 shows the baseline and clinical characteristics of patients with enterococcal BSI compared by groups. Patients with BSI due to *E. faecium* were more likely to have received previous antibiotics (mainly carbapenems), previous antifungal prophylaxis and previous blood transfusion. Likewise, they had more prolonged neutropenia than did patients with *E. faecalis* BSI, and were more likely to have a concomitant infection. In addition, there was a trend in the former group of patients towards haematological malignancy as the most frequent underlying disease, and for there to be a venous catheter in place. The most frequent origin of BSI was an endogenous source in 38% of cases (41% in the *E. faecium* group vs 34% in the *E. faecalis* group), followed by catheter infection in 15% of cases (15% vs 16%, respectively) and cholangitis in 13% (15% vs 10.5%, respectively). BSI originating in the urinary tract tended to be more frequent in the group of *E. faecalis*, whereas neutropenic enterocolitis tended to be more frequent in the *E. faecium* group. After applying a logistic regression model the only variable found to be an independent risk factors for *E. faecium* acquisition was previous use of carbapenems (OR 10.24; 95% CI, 1.35-77.66).

**Table 1:** Baseline and clinical characteristics of all episodes of enterococal bacteraemia and risk factors for *E. faecium* acquisition.

	***E. faecalis***	***E. faecium***	***p***	**Adjusted OR**	***p***
**Characteristic**	**n=38 (%**)	**n=54 (%**)		**(95%CI**)	
**Male sex**	27 (71)	32 (59)	0.27	1.70 (0.29-9.81)	0.55
**Age (yrs, median, range**)	61 (26-78)	59 (21-83)	0.15	0.99 (0.94-1.05)	0.98
**Underlying disease**			0.074		
**Solid tumour**	15 (39.5)	12 (22)			
**Haematological malignancy**	23 (60.5)	42 (78)			
**Haematopoietic stem cell transplant (HSCT**)	2 (5)	7 (13)	0.29		
**Type of HSCT**					
**Autologous**	1	1	0.41		
**Allogeneic**	0	4	-		
**Dual**	1	2	-		
**Graft-versus-host disease**	0	2 (4)	0.50		
**Comorbidities**	16 (42)	21 (39)	0.83		
**Diabetes mellitus**	6 (16)	8 (15)	1.00		
**Chronic obstructive pulmonary disease**	2 (5)	1 (2)	0.56		
**Chronic heart disease**	7 (18)	7 (13)	0.56		
**Chronic renal failure**	1 (3)	3 (6)	0.64		
**Chronic liver disease**	2 (5)	1 (2)	0.56		
**Neutrophil count < 500**	19 (50)	35 (65)	0.19		
**Previous days with neutropenia (< 500**)** (median, range**)	8 (0-35)	11 (1-60)	0.028	0.99 (0.92-1.07)	0.95
**MASCC ≥ 21**	9 (56)	17 (55)	1.00		
**Chemotherapy (within 1 month**)	23 (60.5)	41 (76)	0.16		
**Radiotherapy (within 1 month**)	1 (3)	4 (7)	0.40		
**Corticosteroid therapy (within 1 month**)	18 (47)	19 (35)	0.28		
**Antifungal prophylaxis**	12 (32)	32 (59)	0.011	1.31 (0.20-8.35)	0.77
**Previous antibiotic therapy (within 1 month**)	24 (63)	52 (96)	<.001	10.24 (1.35-77.66)	0.024
**Carbapenems**	3 (12,5)	27 (52)	0.001		
**β-lactam + β-lactam inhibitor**	5 (21)	18 (35)	0.28		
**Cephalosporin**	14 (58)	29 (56)	1.00		
**Quinolone**	2 (8)	12 (23)	0.20		
**Aminoglycoside**	1 (4)	1 (2)	0.53		
**Glycopeptide**	5 (21)	10 (19)	1.0		
**Severe mucositis (grade III-IV**)	3 (8)	10 (18.5)	0.22		
**Urinary catheter**	10 (26)	11 (21)	0.61		
**Venous catheter**	28 (74)	48 (89)	0.058		
**Central venous catheter**	15 (39.5)	28 (52)	0.29		
**Previous hospital admission (within 3 months**)	16 (42)	26 (49)	0.53		
**Previous ICU admission (within 3 months**)	5 (13)	7 (13)	1.00		
**Previous episode of bacteremia**	10 (26)	20 (38)	0.27		
**Previous blood transfusion (within 5 days**)	10 (28)	26 (51)	0.046	0.57 (0.12-2.6)	0.48
**Concomitant infection**	4 (11)	15 (29)	0.041	8.4 (0.80-88.33)	0.076
**Axillary temperature ≥ 38 C**	35 (92)	46 (85)	0.51		
**Polymicrobial bacteraemia**	9 (24)	11 (20)	0.79		
**Persistent bacteraemia**	4 (11)	7 (15)	0.75		
**Shock at presentation**	4 (10.5)	2 (4)	0.22		
**Source of bacteraemia**					
**Endogenous source**	13 (34)	22 (41)	0.66		
**Gastrointestinal tract**	3 (8)	3 (6)	0.68		
**Neutropenic enterocolitis**	1 (3)	8 (15)	0.076		
**Cholangitis**	4 (10.5)	8 (15)	0.75		
**Urinary tract**	5 (13)	1 (2)	0.078		
**Catheter-related**	6 (16)	8 (15)	1.00		
**Unknown**	5 (13)	3 (6)	0.26		

### Microbiological studies

All *E. faecium* isolates were vancomycin and teicoplanin susceptible. Only two strains (2.9%) of the 54 isolates were ampicillin susceptible with MICs ≤1 µg/mL. During the study period 97.1% of strains showed high-level resistance to ampicillin, and the rates of high-level resistance to streptomycin and gentamycin were 74.6% and 32.8%, respectively. Quinolone resistance accounted for 88.8% of the isolates, with MICs ≥4 µg/mL.

**Figure 1:**
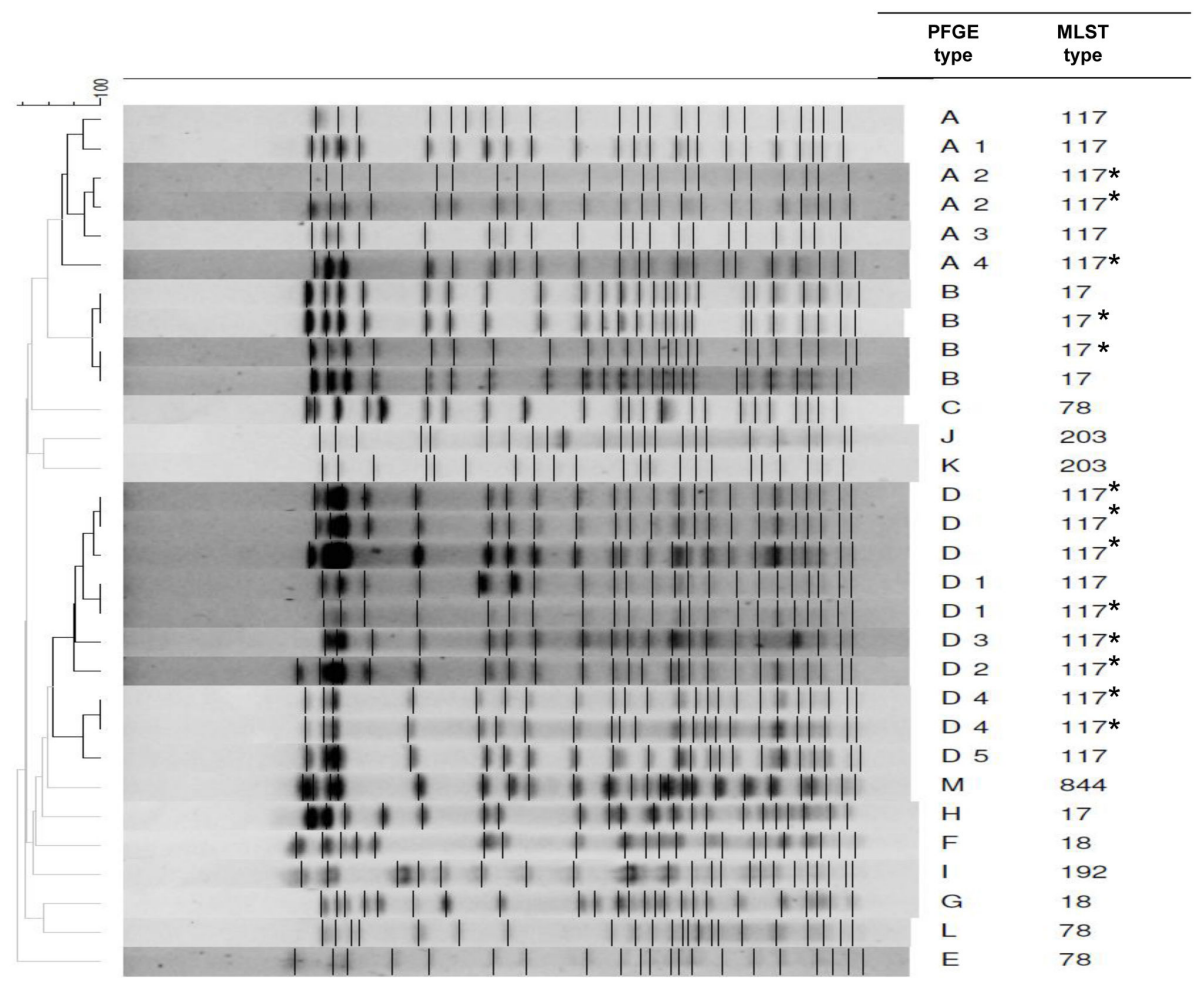
Cluster analysis of Pulsed-field Gel Electrophoresis (PFGE) *Sma*I macrorestriction fragments of the 30 *Enterococcus faecium* strains followed by multilocus sequence typing (MLST) data. MLST was inferred in those strains that were marked with an asterisk. For dendrogram construction, optimization and band position tolerance were set at 0.5% and 1.0% respectively. The cut-off value to define the PFGE patterns was set at 80% similarity.

Among 30 ampicillin-resistant *E. faecium* strains available for genotyping, 13 PFGE patterns were obtained corresponding to 7 STs (Figure 1). ST117 was dominant, accounting for 16 isolates (53.3%) belonging to PFGE type D (n=10; 62.5%) and PFGE type A (n=6; 37.5%). Five isolates (16.7%) belonged to ST17 of PFGE type B (n=4) and H (n=1), three isolates (10%) belonged to ST 78, two (6.7%), to ST18, two (6.7%) to ST203 and one to ST192. A single isolate belonged to a new ST844 (Table S1). Considering the new classification recently proposed by Willems and co-workers, 5 isolates belonged to lineage 17 (ST17), two isolates belonged to lineage 18 (ST18) and 23 isolates belonged to lineage 78 (ST78, ST117, ST192, ST203 and ST844) [6]. From 2006 to 2009, one isolate out of eight available for genotyping belonged to ST117 (year 2009). In contrast, from 2010 to 2012, 15 out of 22 isolates belonged to ST117, with PFGE patterns D (n=10) and A (n=5). The allelic profiles for all different STs are detailed in Table S1.

Antibiotic treatment and patient outcomes are detailed in Table 2. The large majority of patients received empirical antibiotic therapy (91%). The most frequently antibiotic used was a glycopeptide (42% of cases), followed by third- and fourth-generation cephalosporins (29%), β-lactam plus β-lactam inhibitors (26%) and carbapenems (25%). More patients with *E. faecalis* BSI received a combination of a β-lactam plus β-lactam inhibitor empirically, whereas more patients in the *E. faecium* group were given a glycopeptide. Patients with *E. faecium* BSI were more likely to have received inadequate initial empirical antibiotic therapy than were patients with *E. faecalis* BSI, and time to adequate empirical antibiotic therapy was also longer in the former group. No significant differences were found between the two groups regarding other outcomes such as early and overall mortality rates.

**Table 2:** Antibiotic therapy and outcome of patients with enterococal bacteraemia compared by groups.

	***E. faecalis***	***E. faecium***	***p***
	**n=38 (%**)	**n=54 (%**)	
**Empirical antibiotic therapy**	35 (92)	49 (91)	1.00
**Inadequate empirical antibiotic therapy**	9 (24)	24 (44)	0.041
**β-lactam + β-lactam inhibitor**	13 (37)	9 (18)	0.054
**Cephalosporin**	11 (31)	13 (26.5)	0.63
**Carbapenem**	8 (23)	13 (26.5)	0.80
**Glycopeptide**	10 (29)	25 (51)	0.046
**Quinolone**	1 (3)	2 (4)	1.00
**Aminoglycoside**	5 (14)	5 (10)	0.73
**Combination therapy**	8 (23)	6 (12)	0.24
**Days to adequate empirical antibiotic therapy (median, range**)	0 (0-6)	1 (0-4)	0.036
**Intensive care unit admission**	5 (13)	7 (13)	1.00
**Mechanical ventilation**	2 (6)	4 (8)	1.00
**Overall case-fatality rate (30 days**)	10 (26)	16 (30)	0.72
**Early case-fatality rate (48 hours**)	1 (3)	3 (6)	0.64

Risk factors associated with overall case-fatality in the cohort of patients with enterococcal BSI are shown in Table 3. The use of current corticosteroids, shock at presentation, intensive care unit (ICU) admission, mechanical ventilation and unknown source of BSI were the variables most frequently found in the group of patients who died. However, after applying a logistic regression model the only variables found to be independent risk factors for overall case-fatality were the current use of corticosteroids (OR 4.18; 95% CI, 1.34-13.01) and ICU admission (OR 9.97; 95% CI, 1.96-50.63). BSI due to *E. faecium* was not identified as a risk factor for overall mortality.

**Table 3:** Risk factors for overall mortality (30 days) in the cohort of patients with enterococcal bacteraemia.

	***E. faecalis***	***E. faecium***	***p***	**Adjusted OR**	***p***
**Characteristic**	**n=38 (%**)	**n=54 (%**)		**(95%CI**)	
**Male sex**	43 (65)	16 (61.5)	0.81	1.23 (0.38-3.89)	0.72
**Age (yrs, median, range**)	61 (26-78)	59 (21-83)	0.15	0.99 (0.95-1.03	0.65
**Underlying haematological disease**	49 (74)	16 (61.5)	0.30		
**Neutropenia (<500**)	40 (61)	14 (54)	0.64		
**Corticosteroids**	20 (30)	17 (65)	0.004	4.18 (1.34-13.01)	0.013
**Shock at presentation**	1 (1.5)	5 (19)	0.006	3.43 (0.27-43.70)	0.34
**Inadequate empirical antibiotic therapy**	26 (39)	7 (27)	0.33		
**Intensive care unit admission**	3 (4.5)	9 (35)	<.001	9.97 (1.96-50.63)	0.006
**Mechanical ventilation**	1 (2)	5 (22)	0.005		
**Unknown source of bacteraemia**	3 (4.5)	5 (19)	0.038	3.99 (0.68-23.40)	0.12
***E. faecium* bacteraemia**	38 (58)	16 (61.5)	0.81		

## Discussion

We observed a dramatic increase in the incidence of ampicillin-resistant, vancomycin-susceptible *E. faecium* BSI in patients with cancer over the study period. Historically, *E. faecalis* was the responsible for the large majority of all clinical enterococcal infections, with *E. faecium* being less frequently isolated. However, in the late 1990s the ratio of *E. faecalis* to *E. faecium* infections in the United States shifted in favour of *E. faecium*, while in Europe the first reports of increased numbers of infections due to *E. faecium* were published in the mid-1990s [1,9,13]. Despite this, information regarding BSI caused by vancomycin-susceptible *E. faecium* in immunosuppressed cancer patients is scarce in the literature, and all the articles published to date are retrospective studies [11,14,15,22]. Thus, our study is the first prospective analysis of a cohort of cancer patients with vancomycin-susceptible *E. faecium* BSI to be conducted at a time when enterococcal infections are gaining importance worldwide, both in terms of dissemination and antimicrobial resistance. It is also the first study to describe how vancomycin-susceptible *E. faecium* outnumbers *E. faecalis* in this high-risk population of patients with BSI in our geographical area.

Although several studies have focused on risk factors for vancomycin-resistant enterococcal infection or colonization [23,24], little is known about risk factors for BSI due to vancomycin-susceptible *E. faecium*. In our study we identified previous use of carbapenems as the only independent risk factor associated with *E. faecium* BSI. Since the early research of Boyce et al., which related ampicillin-resistant enterococci to the use of imipenem, several authors have demonstrated the association of ampicillin-resistant enterococci with β-lactams [9-11,16,25]. Antibiotics may facilitate colonization and infection by depleting the gastrointestinal tract of its normal anaerobic flora and by selecting enterococci due to limited bactericidal activity against these organisms. The use of broad spectrum β-lactams (including carbapenems) in patients with cancer and frequently-associated febrile neutropenia is very common in clinical practice. Furthermore, the emergence of multidrug-resistant organisms (especially extended-spectrum β-lactamase-producing *Enterobacteriaceae*) among cancer patients in our centre often forces us to use carbapenems as the treatment of choice [26]. A judicious use of antibiotics is therefore needed in order to avoid the development and dissemination of bacterial resistance.

Our study shows that all the enterococci isolates remained susceptible to glycopeptides. Although vancomycin resistance has become an emerging health problem worldwide, it is less important in Europe than in the United States, and as yet it does not seem to be a problem in our geographical area [1,13]. However, the emergence of *E. faecium* is of potential concern as it is more commonly associated with vancomycin resistance than are the other enterococci [27]. The large majority of *E. faecium* isolates showed a high level of resistance to ampicillin, and only two strains were ampicillin susceptible. Notably, the two patients carrying the two susceptible strains had not received carbapenems previously.

PFGE and MLST analysis of 30 available *E. faecium* isolates showed that all isolates belonged to the three major recently described hospital-associated *E. faecium* lineages (17, 18 and 78) [6]. Of note, the ST117 (lineage 78) was especially frequent in the last three years, which may explain, at least partially, the emergence of *E. faecium* in our centre. This finding is in line with recent molecular epidemiological studies that have identified these three lineages (formerly CC17) as being responsible for the worldwide emergence of ampicillin-resistant *E. faecium*. These three lineages have adapted extremely well to the hospital environment, including the acquisition of ampicillin resistance and the *esp* virulence gene, which is associated with biofilm formation. Therefore, these lineages have become the leading cause of hospital-acquired *E. faecium* infections and outbreaks [2-4,28]. The partial replacement of ampicillin-susceptible *E. faecalis* by hospital-associated lineages of ampicillin-resistant *E. faecium* is worrying, since it may set the stage for the emergence of vancomycin-resistant *E. faecium*.

There is controversy in the literature regarding the association between *E. faecium* infection and mortality. Some authors have reported increased mortality in those patients with BSI due to ampicillin-resistant *E. faecium* [7,10,16]. However, it is still unclear if the increase in mortality actually depends on infection or, rather, whether infection behaves as a marker of the severity of underlying diseases [29]. In our study, and in line with some previous reports, we found no association between *E. faecium* BSI and increased mortality [8,9,14,28]. Interestingly, some in vitro studies have suggested that enterococcal virulence determinants are more frequently found in *E. faecalis* isolates than in *E. faecium* isolates [30]. On the other hand, some studies have reported that *E. faecium* is more often resistant to phagocytosis than is *E. faecalis* [31]. Whether there is a clinically relevant difference in virulence between vancomycin-resistant enterococci and vancomycin-susceptible enterococci, or between different enterococcal species is unknown.

The only variables found to be independent risk factors for mortality in our study were ICU admission and current corticosteroid therapy. ICU admission is associated with severe sepsis and shock, which are known to be risk factors for mortality in patients with BSI [7]. Patients receiving corticosteroid therapy mainly corresponded to debilitated patients with severe uncontrolled underlying disease, who are known to be a risk group for poor outcome.

Patients with *E. faecium* BSI in our study were more likely to receive inadequate empirical antibiotic therapy. Inadequate empirical antibiotic therapy has previously been reported to be associated with mortality, especially in patients with Gram-negative BSI and in those with vancomycin-resistant enterococcal infections [32]. However, this association was not observed in our study. A retrospective study by DiazGranados et al. identified vancomycin-resistance as a risk factor for mortality in neutropenic cancer patients. However, it was not associated with inadequate empirical antibiotic therapy, but rather was attributed to prolonged duration of BSI [15]. Factors influencing mortality among cancer patients are often difficult to asses.

The emergence of *E. faecium* in immunosuppressed patients with cancer is a concern, since there are limited therapeutic options for these organisms. Although new antimicrobials, such as linezolid, daptomycin and quinupristin/dalfopristin, have recently been developed to treat serious enterococcal infections, resistance to these agents has already emerged [33-35].

This study has a number of strengths. It describes the incidence of *E. faecium* BSI in a large cohort of BSI episodes prospectively collected in a specific immunosuppressed high-risk population. Additionally, it provides information regarding the *E. faecium* clonal complexes identified during the study period. However, it also has certain limitations. The small number of patients in the two groups may have prevented us from identifying significant differences between them. Also, as this was a single-centre study the results may have been influenced by local epidemiological variables, thereby limiting their applicability to other settings.

In conclusion, we found a significant increase in vancomycin-susceptible *E. faecium* BSI among cancer patients, especially those treated previously with carbapenems. Clonal complex 17 was responsible for the large majority of *E. faecium* infections, particularly in recent years. The emergence of *E. faecium* among immunosuppressed cancer patients is worrying since there are limited treatment options and it may presage the emergence of vancomycin-resistant enterococci. Addressing this trend for enterococci requires a rational approach that combines infection control with antimicrobial stewardship.

## Supporting Information

Table S1 **Allelic profiles among 17 *E. faecium* blood isolates.**
(DOC)
