# Supplementary material for: Increase in Bloodstream Infection Due to Vancomycin-Susceptible Enterococcus faecium in Cancer Patients: Risk Factors, Molecular Epidemiology and Outcomes
Source: PLoS One. 2013 Sep 19;8(9):e74734. doi: 10.1371/journal.pone.0074734 (PMC3778008; doi:10.1371/journal.pone.0074734)
Supplement: Table S1 — Allelic profiles among 17 E. faecium blood isolates. (DOC) [file pone.0074734.s001.doc]

**Table S1 -** Allelic profiles among 17 *E. faecium* blood isolates

| **MLST** | **Lineage** | **PFGE type** | **Allelic profile^a^** | | | | | | | **No. of isolates** |
| --- | --- | --- | --- | --- | --- | --- | --- | --- | --- | --- |
|  |  |  | ***atpA*** | ***ddl*** | ***gdh*** | ***purK*** | ***gyd*** | ***pstS*** | ***adk*** |  |
| **17** | **17** | B | 1 | 1 | 1 | 1 | 1 | 1 | 1 | 2 |
|  |  | H | 1 | 1 | 1 | 1 | 1 | 1 | 1 | 1 |
| **18** | **18** | F | 7 | 1 | 1 | 1 | 5 | 1 | 1 | 1 |
|  |  | G | 7 | 1 | 1 | 1 | 5 | 1 | 1 | 1 |
| **78** | **78** | C | 15 | 1 | 1 | 1 | 1 | 1 | 1 | 1 |
|  |  | E | 15 | 1 | 1 | 1 | 1 | 1 | 1 | 1 |
|  |  | L | 15 | 1 | 1 | 1 | 1 | 1 | 1 | 1 |
| **117** | **78** | A | 9 | 1 | 1 | 1 | 1 | 1 | 1 | 3 |
|  |  | D | 9 | 1 | 1 | 1 | 1 | 1 | 1 | 2 |
| **192** | **78** | I | 15 | 1 | 1 | 1 | 1 | 7 | 1 | 1 |
| **203** | **78** | J | 15 | 1 | 1 | 1 | 1 | 20 | 1 | 1 |
|  |  | K | 15 | 1 | 1 | 1 | 1 | 20 | 1 | 1 |
| **844** | **78** | M | 15 | 2 | 1 | 1 | 1 | 11 | 1 | 1 |
| **Total No of Isolates** | | | | | | | | | | **17** |

^a^ *adk*, gene coding for adenylate kinase; *atpA*, gene coding for ATP synthase, alpha subunit; *ddl*, gene coding for D-alanine:D-alanine ligase; *gyd*, gene coding for glyceraldehyde-3-phosphate dehydrogenase; *gdh*, gene coding for glucose 6-phosphate dehydrogenase; *pstS*, gene coding for phosphate ATP binding cassette transporter; *purK*, gene coding for phosphoribosylaminoimidazol carboxylase ATPase subunit.
